# 

**Published:** 1894-12

**Authors:** 


### CONTENTS:

### SELECTIONS.

Article I.—Discrimination in Filling Materials. By Dr. J. L. Davenport - - - - - - - - 337
" II.—Our Present Status. By T. B. Welch, M. D. - - 342
" III.—Ideal Bridge Work. By R. A. Holliday, D. D. S. - 345
" IV.—The Treatment and Filling of Pulpless Teeth as Learned from Papers Read at the World's Dental Congress, A. D., 1893. By S. D. Banes, D. D. S. - 350
" V.—Artificial Crowns. By Dr. J. H. Crossland. - - 359
" VI.—Malpractice. - - - - - - - - 362
" VII.—Filling Children's Teeth. By Dr. W. G. A. Bonwill. 369

### EDITORIAL, ETC.

Free Artificial Teeth for Soldiers. - - - - - - - 375
Dental Department of Howard University. - - - - 375

### MONTHLY SUMMARY.

Little Aids in Practice. - - - - - - - - 375

Hints, - - - - - - - - - - - 377

A Nice Appearing Lady Dentist. - - - - - - - 378

Disease of the Antrum. - - - - - - - - - 378

Comparison of Professional Fees. - - - - - - - 379

The Contagion of the Communion Cup. - - - - - 380

Died While Having a Tooth Extracted. - - - - - 381

Alcohol in Shock. - - - - - - - - - 382

Hydrogen Dioxide. - - - - - - - - - 382

